# Self-, other-, and dual-harm during adolescence: a prospective-longitudinal study of childhood risk factors and early adult correlates

**DOI:** 10.1017/S0033291722000666

**Published:** 2023-07

**Authors:** Annekatrin Steinhoff, Laura Bechtiger, Denis Ribeaud, Manuel Eisner, Lilly Shanahan

**Affiliations:** 1 Jacobs Center for Productive Youth Development, University of Zurich, Zurich, Switzerland; 2 Institute of Criminology, University of Cambridge, Cambridge, UK; 3 Department of Psychology, University of Zurich, Zurich, Switzerland

**Keywords:** Adolescence, self-harm, aggression, violence, childhood adversity, mental health, risk factors, longitudinal

## Abstract

**Background:**

Little is known about the childhood antecedents and adult correlates of adolescent dual-harm (i.e. co-occurring self- and other-harm). We examine the longitudinal associations between (a) social and psychological risk factors in childhood and adolescent dual-harm and (b) adolescent dual-harm and social and mental health impairments in early adulthood.

**Methods:**

Participants (*N* = 1482) are from a prospective longitudinal community-representative study. Dual-, self-, and other-harm were self-reported at ages 13, 15, and 17. Social and psychological risk factors in childhood were assessed between 7 and 11; early adult correlates at age 20. Groups with dual-harm, self-harm only, other-harm only, and no harm were compared.

**Results:**

Between 13 and 17, 7.2% of adolescents reported dual-harm (self-harm only: 16.2%; other-harm only: 13.3%). Some childhood risk factors (e.g. sensation-seeking, parental divorce, victimization by peers) characterized all harm groups; others were common to the dual- and self-harm (anxiety/depressive symptoms, relational aggression) or dual- and other-harm groups only (low self-control, substance use, delinquency). Adolescents with dual-harm had reported more physical aggression and harsh parenting, and lower school bonding in childhood than any other group. In early adulthood, they reported more anxiety/depressive symptoms, psychopathy symptoms, homicidal ideations, delinquency, and victimization experiences than any other group.

**Conclusions:**

Adolescent dual-harm follows psychological problems and social disconnection in childhood and signals risk of psychopathology and isolation in early adulthood. To curb the burden from dual-harm, interventions must target adolescents, families, peer networks, and school environments. Differentiating youth with dual-harm from those with single-harm is important for developing personalized treatments.

Harm against self and others ranks among the top public health challenges facing young people in the Western world (Krug, Dahlberg, Mercy, Zwi, & Lozano, 2002; Monto, McRee, & Deryck, 2018; Wolf, Gray, & Fazel, 2014) and inflicts substantial costs on individuals and societies (Tsiachristas et al., 2017; Welsh et al., 2008). In some adolescents, self- and other-harm co-occur, either concurrently or sequentially (Harford, Chen, & Grant, 2016; Richmond-Rakerd et al., 2019). However, research has typically studied self- and other-harm separately, and scientific knowledge about adolescent ‘dual-harm’ (i.e. co-occurrence of self- and other-harm) and its developmental antecedents and subsequent correlates is scarce (O'Donnell, House, & Waterman, 2015; Richmond-Rakerd et al., 2019). As a consequence, comprehensive characterizations of individuals with dual-harm and the long-term risks associated with this behavior are lacking. A better understanding is needed of the childhood risk factors and adult sequelae of adolescent dual-harm, and comparisons with the risk factors and sequelae associated with single-harm, to inform early and personalized interventions that are capable to effectively curb the burden from this behavior.

The current study uses data from a prospective longitudinal community-representative cohort study that assessed participants from childhood into adulthood. We considered a broad range of childhood antecedents and adult correlates of adolescent dual-harm and stringently documented the temporal order of events (i.e. childhood risks, adolescent dual-harm, early adult correlates) to address three key but understudied questions: (1) How prevalent is dual-harm among male and female adolescents from the community? (2) What are the childhood risk factors for dual- *v.* single-harm, from the domains of psychopathology (e.g. internalizing and externalizing problems, lack of self-control, substance use) and social experiences (e.g. adversity in the family, peer networks, and school)? (3) Is adolescent dual-harm associated with specific mental health and social impairments in early adulthood?

## Childhood risk factors of dual- and single-harm

Self-harm (e.g. self-cutting) and other-harm (e.g. assault) are common in adolescence (Brown & Plener, 2017; Krug et al., 2002; Monto et al., 2018). Although they involve similar behaviors (e.g. hitting, cutting), self-harm typically occurs in social isolation (Hooley & Franklin, 2017; Nock, 2009), whereas other-harm by definition involves another person. Studies of self- and other-harm converge on several risk factors (e.g. maltreatment and victimization experiences, negative emotions, externalizing and internalizing psychopathology) (Brown & Plener, 2017; Duke, Pettingell, McMoris, & Borowsky, 2010; Farrington, 2018; Nock, Joiner, Gordon, Lloyd-Richardson, & Prinstein, 2006; O'Donnell et al., 2015; Ribeaud & Eisner, 2010; Yen et al., 2010) and diverge on others. For example, female sex is typically a risk factor for self-harm (Bresin & Schoenleber, 2015) and male sex is a risk factor for other-harm (Farrington, 2018); low self-control more typically precedes other-harm than self-harm (Farrington, 2018; Janis & Nock, 2009; Piquero, MacDonald, Dobrin, Daigle, & Cullen, 2005). Overall, however, evidence suggests that risk factors for self- and other-harm often overlap, but because these findings are mostly from separate studies, they are difficult to compare and do not explain why some adolescents engage in other-harm, while others engage in self-harm.

Moreover, dual-harm has typically been neglected. Although dual-harm combines two forms of harm, the associated risk factors could be different from those associated with the individual forms of harm. Cumulative risk research shows that adolescents with more problem behaviors typically come from backgrounds with multiple risks (Appleyard, Egeland, Van Dulmen, & Sroufe, 2005). Two prospective longitudinal community- and population-based studies suggested that adverse childhood experiences (e.g. exposure to violence, poly-victimization) were associated with an increased risk of subsequent dual-harm (Carr et al., 2020; Richmond-Rakerd et al., 2019). One of these studies also examined children's psychological risk factors for dual-harm and found that low self-control was particularly prevalent among adolescents with dual-harm compared to those with self- or other-harm only (Richmond-Rakerd et al., 2019). However, some other factors that are known to increase the risk of harm have not been studied in relation to dual-harm, such as certain forms of childhood psychopathology (e.g. externalizing problems, childhood substance use) and social risks (e.g. low school bonding).

## Early adult correlates of dual- and single-harm

The literature on comorbidity suggests that individuals facing two or more problem behaviors tend to have more serious and impairing problems in later life than those facing a single or no psychological problems (Crawford et al., 2008; Sourander et al., 2007). To understand whether this is true in the case of co-occurring self- and other-harm, and to detect the specific long-term impairments associated with dual-harm, a comparison of the psychological and social functioning of early adults with prior (i.e. adolescent) dual- *v.* single-harm is needed.

Research has shown that individuals with adolescent self- or other-harm often face significant psychological and social impairments later in life. For example, adolescent other-harm precedes a high risk of psychiatric symptoms, substance use, and several social impairments (e.g. delinquency, financial and work-related problems) years and decades later (Moffitt, Caspi, Harrington, & Milne, 2002; Odgers et al., 2008). Adolescent self-harm also predicts later substance use and mental health and social impairments (Borschmann et al., 2017; Groschwitz et al., 2015; Sinclair, Hawton, & Gray, 2010); less is known about adult social problem behaviors (e.g. delinquency) associated with adolescent self-harm. Some evidence suggests that adolescents with dual-harm are at higher risk of poor subsequent well-being (Richmond-Rakerd et al., 2019) and premature mortality (Steeg et al., 2019) than those with single-harm. However, no longitudinal study has examined mental health and social impairments of adolescents with dual-harm past age 18.

## The current study

In summary, research is currently lacking a comprehensive developmental characterization of adolescents with dual-harm, which considers both childhood risk factors and adult correlates. To rectify this gap in research, we compared childhood antecedents and early adult sequelae of adolescent dual-harm with those of adolescent single- and no harm, using data from a large-scale prospective longitudinal study. Harm was measured repeatedly at ages 13–17. We assessed putative developmental antecedents at ages 7–11 and putative early adult correlates at age 20, thereby establishing a developmental timeline from childhood to early adulthood. To obtain a comprehensive characterization of individuals with self-, other-, dual-, and no harm, a broad set of developmental antecedents and outcomes was assessed. We examined several antecedents and outcomes that have been identified in prior research (i.e. victimization experiences, low self-control, any substance use) and additional others, that have not been addressed in previous dual-harm research despite being closely related to self- or other-harm (e.g. externalizing psychopathology, early onset substance use, low school bonding, perceived social exclusion, delinquency). We also included homicidal ideations, which prior dual-harm research has not addressed but which may mirror suicidal ideations as other-harm mirrors self-harm.

## Methods

### Sample and procedures

Data came from six waves of the ongoing longitudinal *Zurich Project on Social Development from Childhood to Adulthood* (*z-proso*; Ribeaud, Murray, Shanahan, Shanahan, & Eisner, 2022). Participants were selected using cluster-stratified randomized sampling. In 2004, 1675 children from 56 primary schools were randomly selected from 90 public schools in the city of Zurich, Switzerland's largest city. Stratification was performed, accounting for school sizes and socio-economic background of the school districts. The sample was largely representative of first-graders attending public school in Zurich, and participants were followed until 2018, when they were 20 years old.

We use data collected from age 7 onwards from respondents who participated at least once between ages 13 and 17, when self- and other-harm were assessed (*N* = 1482, 52% male). Consistent with Switzerland's immigration policies and Zurich's diverse population, the adolescents' parents had been born in over 80 different countries (50% of adolescents had two parents born abroad). The majority of adolescents were born in Switzerland (91%). Parental educational background was diverse; 26% of families had at least one parent with a university degree. The mean household *International Socio-Economic Index of Occupational Status* (ISEI) was 45.74 (s.d. = 19.24). This is an internationally comparable index of socio-economic status based on occupation-specific income and required educational level; scores ranged from 16 (e.g. unskilled worker) to 90 (e.g. judge).

The study complies with national and international ethics standards and was approved by the responsible ethics committee at the Faculty of Arts and Social Sciences, University of Zurich. Adolescents provided written informed consent for their participation; until age 15, parents could choose not to have their child participate. Data were collected in groups of 5–25 participants in classroom-based settings with paper-and-pencil questionnaires up to age 17 and in a computer laboratory with computer-administered surveys at age 20. Surveys typically took approximately 90 min to complete. Participants received a cash incentive, which increased from approximately $30 at age 13 to $75 at age 20.

### Variables

*Self-harm* was self-reported at ages 13, 15, and 17 using one item. Adolescents indicated how often they had injured themselves on purpose during the previous month. Example behaviors provided were ‘cut my arm’, ‘tore open wounds’, ‘hit my head’, and ‘tore out my hair’. Answers were recorded on a five-point scale (1 = ‘never’, 2 = ‘rarely’, 3 = ‘sometimes’, 4 = ‘often’, 5 = ‘very often’) and were dichotomized (0 = no self-harm, 1 = any self-harm) (Steinhoff et al., 2021).

*Other-harm* (at ages 13, 15, and 17) was assessed using an indicator of assault taken from a broader delinquency scale with 19 items altogether, which asked about several relatively mild (e.g. stealing at home, vandalism) and severe (e.g. shoplifting >50 CHF, drug dealing, assault) acts of delinquency (adapted from Eisner, Manzoni, & Ribeaud, 2000; Ribeaud & Eisner, 2009; Wetzels, Enzmann, Mecklenburg, & Pfeiffer, 2001). To assess the incidence of assault, adolescents indicated whether they had ‘purposely hit, kicked, or cut someone, and injured him or her in the process’ in the previous year and, if so, how often (open question). Potential victims included familiar and unfamiliar peers and adults. We used a dichotomous variable (0 = no other-harm and 1 = at least one incidence of other-harm).

*Dual-harm* was coded when adolescents reported both self- and other-harm, either at the same or different assessments between ages 13 and 17. Adolescents who reported self-harm at least once between 13 and 17 and did not report other-harm at any time were assigned to a ‘self-harm only’ group. Conversely, adolescents who reported other-harm at least once between 13 and 17 and did not report any self-harm were assigned to an ‘other-harm only’ group. Those who reported no self- or other-harm were assigned to a ‘no harm’ group. This coding scheme allowed us to distinguish reasonably-sized groups with different types of harm across the adolescent period, which is also comparable to the approach taken in previous research on dual- *v.* single-harm (Richmond-Rakerd et al., 2019).

*Childhood risk factors* (ages 7–11) are described in online Supplementary Table S1. The variables cover the domains of personality, psychopathology, and behavior (i.e. sensation-seeking, anxiety/depressive symptoms, lack of self-control, relational and physical aggression, substance use, delinquency), and adverse social experiences (i.e. parental divorce, harsh parenting, lack of parental involvement, bullying and violent victimization by peers, low school bonding). Compared to our narrow conceptualization of other-harm as a form of aggression that inflicts injury upon others, the childhood physical aggression measure included milder forms of violence, and did not explicitly assess whether the child had injured somebody. Although there is some conceptual overlap between physical aggression and other-harm, we examined the potentially milder physical aggression variable as a precursor of adolescent harm. Physical aggression, even in its milder forms, is one of the more easily observable and identifiable indicators of risk. Thus, if there is a link between earlier physical aggression and membership in the single- or dual-harm groups, this would be important to know for future risk screening and prevention efforts.

*Early adult mental health and social impairments* (age 20) are also described in online Supplementary Table S1. We used all constructs that were included in the childhood risk assessment, except sensation-seeking and family and school-related risks. Symptoms of psychopathy, suicidal and homicidal ideations, and perceived social exclusion were also included to represent important putative correlates of self-/other-/dual-harm that were only assessed after the onset of adolescence.

*Socio-demographic* control variables, including sex, parental education background, child's educational level at age 13, and parental migration background, were coded dichotomously (for details, see online Supplementary material).

### Analytic strategy

First, we identified the prevalence of dual- and single-harm between ages 13 and 17 and associated sex differences. Second, we investigated childhood risk factors associated with adolescent dual-/single-/no harm by comparing group-specific prevalence (for dichotomous variables) and standardized *z*-scores (for continuous variables). To assess effect sizes adjusted for socio-demographic differences, we conducted multinomial regression analyses, in which the groupings were the dependent variable and sex, parental migration and educational background, and child's educational level at age 13 were the control variables. Third, we examined early adult correlates of dual-/single-/no harm using a similar strategy, with adolescent dual-/single-/no harm as independent variables in the regression models. To determine significance, we used *p* < 0.05 in linear regression models and the 95% confidence interval (CI) in logistic regression models. Consistent with our goal of providing a broad and novel developmental characterization of youth with dual-harm, and considering the caveats associated with multiple testing, we primarily focus on patterns of results (e.g. the spectrum of risk factors associated with particular types of harm; commonalities between risk factors linked with harm) in the interpretation of findings, whereas single significant coefficients are regarded as exemplifications of such patterns. Although our hypotheses were directional in nature (i.e. higher levels of risk are associated with dual- and single-harm compared to no harm, and with dual-harm compared to single-harm), we used the more conservative approach of two-tailed testing.

To reduce potential bias due to attrition, we conducted multiple imputation of missing data prior to the multivariate analyses (Schafer & Graham, 2002). Descriptive analyses were conducted in SPSS, and imputation and regression models were specified in Mplus V8. We used maximum likelihood robust estimation.

## Results

### Prevalence of adolescent dual- and single-harm

**Fig. 1.**
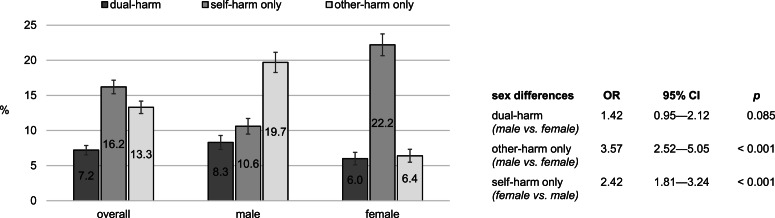
Prevalence of dual-harm, self-harm only, and other-harm only between ages 13 and 17 and respective sex differences (*N* = 1482).

Overall, one in 14 adolescents (*n* = 107/1482; 95% CI of prevalence 6.0–8.7%) reported dual-harm between ages 13 and 17, one in six reported self-harm only (*n* = 240/1482; 95% CI 14.4–18.2%), and one in seven reported other-harm only (*n* = 197/1482; 95% CI 11.6–15.1%) (see Fig. 1). Membership in the dual-harm group did not differ by sex. Males had a higher risk of other-harm only than females; females were more likely to report self-harm only than males. For associations between harm and other socio-demographics, see online Supplementary material.

### Childhood risk factors

**Fig. 2.**
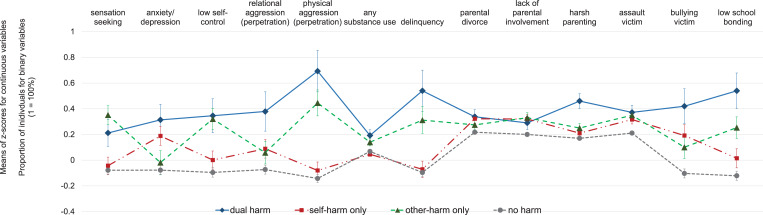
Childhood risk profiles of groups with dual-, single-, and no harm between ages 13 and 17.

Adolescents with dual-harm between ages 13 and 17 scored higher on almost all childhood risk factors considered here compared to those with no harm (for bivariate contrasts, see Figure 2; for adjusted contrasts, see Table 1). Self- and other-harm only were each associated with a unique subset of risk factors (Fig. 2; online Supplementary Table S2). Differences between youth with dual- *v.* single-harm were found in both the psychopathology and social domains. Specifically, youth with dual-harm were more physically aggressive and experienced more harsh parenting and less school bonding compared to youth in both single-harm groups (Table 1). Compared to youth with self-harm only, those with dual-harm also had lower self-control, more substance use, and more delinquency. Compared to youth with other-harm only, those with dual-harm showed more relational aggression and experienced bullying victimization more frequently. All three harm groups scored higher than the no harm group for sensation-seeking, physical aggression, prevalence of parental separation, and victimization by peers, and lower for school bonding (with regard to sensation-seeking and physical aggression, it should be noted that the difference between the self-harm only and no harm groups was only significant in the adjusted model, and stepwise modeling suggested that these effects emerged when sex was entered into the model). A direct comparison between the two single-harm groups (online Supplementary Table S2) identified childhood lack of self-control, physical aggression, and delinquency as distinctive risk factors for other-harm only.

**Table 1.** Adjusted associations between childhood risk factors (independent variables) and dual-harm between ages 13 and 17 (dependent variable): results from multinomial regression analyses [OR (95% CI)] controlling for sex, parental educational and migration background, and child's educational level at age 13

Childhood risk factors: independent variables	Dual-harm *v*. no harm	Dual-harm *v*. self-harm only	Dual-harm *v*. other-harm only
Sensation-seeking	**1.29** (1.02–1.63)	1.06 (0.81–1.39)	0.98 (0.74–1.29)
Anxiety/depression	**1.50** (1.18–1.89)	1.17 (0.90–1.52)	1.29 (1.00–1.68)
Lack of self-control	**1.63** (1.28–2.06)	**1.39** (1.07–1.81)	1.11 (0.84–1.47)
Relational aggression	**1.45** (1.19–1.76)	1.17 (0.93–1.46)	**1.30** (1.03–1.63)
Physical aggression	**2.12** (1.72–2.62)	**1.67** (1.29–2.16)	**1.27** (1.01–1.60)
Substance use	**3.00** (1.60–5.63)	**3.02** (1.26–7.20)	1.47 (0.69–3.14)
Delinquency	**1.69** (1.39–2.06)	**1.51** (1.21–1.88)	1.21 (0.98–1.49)
Parental divorce	**2.16** (1.24–3.76)	1.42 (0.75–2.69)	1.39 (0.73–2.62)
Lack of parental involvement	1.39 (0.84–2.30)	0.74 (0.42–1.28)	0.96 (0.52–1.77)
Harsh parenting	**3.54** (2.15–5.84)	**2.58** (1.50–4.44)	**2.19** (1.24–3.87)
Assault victimization	**2.16** (1.31–3.56)	1.21 (0.71–2.07)	1.22 (0.68–2.17)
Bullying victimization	**1.57** (1.29–1.91)	1.12 (0.89–1.40)	**1.31** (1.05–1.64)
Lack of school bonding	**1.89** (1.50–2.38)	**1.57** (1.21–2.05)	**1.36** (1.05–1.77)

A separate model was specified for each risk factor. Bold print indicates significant effects (*p* < 0.05).

### Early adult correlates

**Fig. 3.**
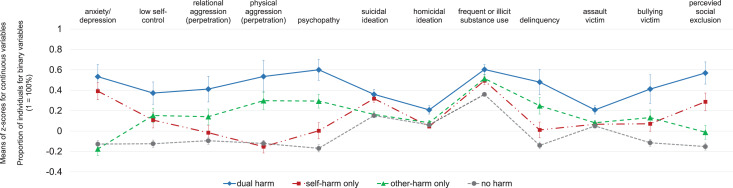
Early adulthood mental health and social impairments of groups with dual-, single-, and no harm between ages 13 and 17.

At age 20, individuals with prior dual-harm had higher scores than those with no harm for all the mental health and social impairments considered (Fig. 3, Table 2). Young adults with prior dual-harm had more anxiety/depressive symptoms, psychopathy symptoms, homicidal ideations, and delinquency and were more frequently victimized by others than any other group. They also reported more relational and physical aggression than those with self-harm only and had more suicidal ideations and felt more socially excluded than those with other-harm only. A direct comparison of the two single-harm groups (online Supplementary Table S3) revealed several differences: those with self-harm only had more anxiety/depressive symptoms and suicidal ideations, less physical aggression, and felt more socially excluded than those with other-harm only.

**Table 2.** Adjusted associations between dual-/single-/no harm (ages 13–17; independent variables) and mental health and social impairments in early adulthood (age 20; dependent variables): results from linear regression analyses (a: *β*, *p*) and binary logistic regression analyses (b: OR, 95% CI) controlling for sex, parental educational and migration background, and child's educational level at age 13

Early adulthood correlates: dependent variables	Dual-harm *v*. no harm	Dual-harm *v*. self-harm only	Dual-harm *v*. other-harm only
Anxiety/depression (a)	**0.19** (<0.001)	**0.08** (0.044)	**0.15** (<0.001)
Lack of self-control (a)	**0.13** (<0.001)	0.05 (0.138)	0.07 (0.083)
Relational aggression (a)	**0.12** (0.001)	**0.08** (0.032)	0.07 (0.062)
Physical aggression (a)	**0.18** (<0.001)	**0.16** (<0.001)	0.08 (0.081)
Psychopathy (a)	**0.20** (<0.001)	**0.13** (<0.001)	**0.08** (0.031)
Suicidal ideation (b)	**3.20** (1.95–5.25)	1.15 (0.68–1.95)	**2.84** (1.53–5.29)
Homicidal ideation (b)	**3.21** (1.71–6.02)	**2.45** (1.11–5.41)	**2.12** (1.00–4.46)
Frequent/illicit substance use (b)	**2.74** (1.72–4.37)	1.27 (0.78–2.08)	1.68 (0.97–2.89)
Delinquency (a)	**0.17** (<0.001)	**0.09** (0.016)	**0.08** (0.048)
Assault victim (b)	**4.87** (2.64–8.99)	**2.82** (1.35–5.86)	**2.39** (1.18–4.86)
Bullying victim (a)	**0.15** (<0.001)	**0.09** (0.024)	**0.08** (0.040)
Perceived social exclusion (a)	**0.18** (<0.001)	0.07 (0.066)	**0.15** (<0.001)

A separate regression model was specified for each correlate. Bold print indicates significant effects (*p* < 0.05).

Results from regression models controlling for the respective childhood levels of the age 20 correlates indicate that youth with dual-harm faced a relative increase in all indicators of psychopathology and social impairments considered here between childhood and early adulthood compared to the no harm group (online Supplementary Table S4). The dual-harm group's relative increase in risk of being the victim of an assault was unique compared to all other groups.

### Sensitivity analysis: inclusion of childhood other-harm

In z-proso, other-harm was already assessed at age 11. These data were not included in our coding of harm groups, because self-harm was not assessed at age 11. Nevertheless, to examine the robustness of our results, we conducted a sensitivity analysis of the patterns of age-20 correlates of prior harm, with age-11 other-harm being included in the coding (childhood precursors were not investigated, because in that case a temporal overlap of the assessments of risk factors and harm could not be avoided). As expected, the prevalence of other-harm only (16.6%) and dual-harm (8.4%) were slightly higher, and the prevalence of self-harm only (15.0%) was a bit lower, compared to the original coding. The early adulthood mental health and social impairment profiles of youth with prior dual- and single-harm were replicated (online Supplementary Fig. S1).

## Discussion

Adolescent self-, other-, and particularly dual-harm cause enormous burdens for individuals, families, and society. Designing well-targeted prevention and intervention mechanisms requires an understanding of the antecedents of dual- and single-harm and their early adult correlates. Based on prospective longitudinal community data spanning the entire period from early adolescence to early adulthood, we show that one in 14 adolescents engaged in dual-harm between ages 13 and 17, which means that, on average, at least one or two students in every school class are affected. Our study reveals that these youth constitute a unique high-risk group.

### Prevalence of single- and dual-harm

The prevalence of dual-harm in the current study is about 1.5 times the prevalence reported in a recent UK community study (Richmond-Rakerd et al., 2019). This difference in prevalence may be partly explained by differences in operationalization and cultural contexts but could also mirror discrepancies between retrospective and prospective reporting of mental health and adverse experiences (Moffitt et al., 2010; Reuben et al., 2016). The self-harm assessment in the UK study was based on retrospective reports provided in late adolescence (when self-harm typically ceases). In contrast, we used three repeated assessments during early and mid-adolescence, when self-harm typically peaks (Plener, Schumacher, Munz, & Groschwitz, 2015; Steinhoff et al., 2021). Thus, our assessment may have been less affected by recall bias, resulting in higher rates.

Consistent with previous research on self-harm or other-harm, respectively, females had a higher risk than males of engaging in self-harm only (Bresin & Schoenleber, 2015) and males had a higher risk of engaging in other-harm only (Farrington, 2018). Dual-harm was not sex-differentiated, although there was a weak trend indicating that males had a higher risk of being in the dual-harm group than females.

### Childhood risk factors of dual- and single-harm

Childhood risk factors associated with adolescent dual-harm include psychopathology and social impairments, which indicates that prevention measures may be most promising when they target children's personal resources and adversities in several social contexts (e.g. family, peers, school). This finding confirms and extends the existing evidence (Carr et al., 2020; Richmond-Rakerd et al., 2019) by adding more childhood risk factors that were all prospectively assessed. The latter is important, since research on the long-term effects of childhood risk factors has shown that prospective assessments of risk factors typically yield more reliable risk predictions than retrospective assessments (Reuben et al., 2016).

Our analyses reveal several factors that increase the risk of any form of harm to similar degrees, including childhood sensation-seeking, parental divorce, and violent victimization by peers. Channeling children's inclination to take risks toward healthy behaviors, increasing their capacities to cope with stressful events such as parental separation, and preventing peer violence may therefore be promising priority targets for general intervention programs.

Our analyses also show that comorbidity and co-occurrence of several risks must be considered, particularly in the dual-harm group. For example, high levels of internalizing and externalizing symptoms are combined in this group. In addition, high levels of victimization by peers, combined with children's high levels of aggression toward others, could indicate that youth who bully others and who are also bullied (‘bullied bullies’) are at high risk of dual-harm. This finding adds another severe ‘outcome’ to research showing that ‘bullied bullies’ are at risk of especially problematic mental health and behavioral development (Copeland, Wolke, Angold, & Costello, 2013; Zych et al., 2020).

Our study reveals three *unique* childhood risk factors of dual-harm: very high levels of childhood physical aggression, harsh parenting, and very low school bonding. Common catalysts that could translate these risk factors into problematic youth behavior are a generalized sense of social disconnection and a resultant lack of a sense of belonging, which is vital for human well-being (Baumeister & Leary, 1995). Protecting children from violence in their homes, supporting their sense of belonging within the classroom, and improving their interpersonal skills (Slough, McMahon, & Conduct Problems Prevention Research Group, 2008) may be promising avenues for preventing dual-harm. The very high levels of physical aggression in the dual-harm group could also indicate a particularly early onset of other-harm in this group, which suggests a need for interventions that begin in childhood and target typical risk factors for aggressive behaviors (e.g. poor social skills, hostile attribution bias). Finally, the association between childhood aggression and dual-harm could indicate a higher frequency of violence in the dual-harm group compared to the other-harm only group.

### Early adult correlates of dual- and single-harm

The mental health and social impairments reported by early adults with prior dual-harm are less domain-specific than those reported by young adults with prior single-harm. Unique adult correlates of adolescent dual-harm include very high levels of internalizing symptoms and negative relationship experiences (e.g. being victimized), as well as anti-social attitudes (psychopathy), acts (delinquency), and thoughts (homicidal ideation). This obvious lack of personal and social resources in early adulthood in individuals with a history of dual-harm is concerning given that young people typically face major social and personal transitions during this developmental period (Arnett, 2000), which lay the groundwork for their future adult life.

Our findings from models controlling for childhood risk factors reveal that impaired mental health and social life among youth with dual-harm could result from a sharper relative *decline* (or smaller relative *increase*) of well-being between childhood and early adulthood compared to the other groups. Engaging in both self- and other-harm may hamper the development of healthier coping strategies (Robinson et al., 2019), thereby impairing well-being. Alternatively, youth with dual-harm may face increasing rejection and victimization by others due to the stigma associated with self-harm and the anti-social nature of other-harm. Indeed, we found that victimization by others was a major and unique outcome associated with dual-harm. In turn, these negative social experiences could result in more personal impairments, such as internalizing symptoms, suicidal thoughts, and anti-social views and behaviors (Macmillan, 2001).

### Limitations and future directions

Our study has limitations. First, we distinguished groups with single- and dual-harm based on two items only. Furthermore, our narrow definitions of self- and other-harm as inflicting injury upon oneself and another, respectively, do not include other behaviors on the self-harm (e.g. overdosing on a drug) and other-harm (e.g. robbery with a weapon) spectrums. As a consequence, some adolescents with forms of self- or other-harm not considered here might have been assigned to the no harm group, or a single instead of the dual-harm group. In addition, different time frames were used to assess self- and other-harm (i.e. previous month *v.* previous year), as this secondary data set was not originally conceptualized to combine these measures. Together, these limitations might have led to an underestimation of the prevalence of harm, especially self- and dual-harm. With different operationalizations of harm, the associations between harm groups and their correlates might also vary.

Second, we examined harm from early adolescence onwards, but some adolescents might have initiated self-, other-, or dual-harm already in late childhood. Third, our investigation was based on self-reports of single- and dual-harm, and of their correlates, with few exceptions. The validity of self-reports could be affected by social desirability or perception bias. However, the high prevalence of self- and other-harm in our study indicates that social desirability was not a significant issue. Nevertheless, ideally, future research should include more objective (e.g. experimental, official records) or multi-informant measures (e.g. reported by teachers, parents), to distinguish subjective perceptions of psychopathology and social impairments from actual incidents. Fourth, our analyses do not allow for causal inferences, and it is possible that the many childhood adversities facing adolescents with dual-harm could themselves cause ongoing social and psychological impairments in adulthood. Fifth, our variable-centered analyses of correlates should be complemented by within-person perspectives that identify *intra-individual combinations* of risk factors and outcomes. Sixth, our data are largely representative of youth growing up in an urban area in Switzerland, but it is unknown whether the findings can be generalized to other populations.

Finally, although our study was able to reveal novel evidence of childhood risk factors for and adult correlates of dual-, self-, and other-harm, it was beyond the scope of this paper to also examine the developmental trajectories of harm here. Information on the prevalence of dual- and single-harm at the different assessments and an examination of the continuity of harm and transitions between harm types over time can be found elsewhere (Steinhoff, Ribeaud, Eisner, & Shanahan, under review).

## Conclusion

Self- and other-harm have captured the attention of researchers and clinicians for many years, but youth who engage in both behaviors (i.e. dual-harm) are poorly understood. Our findings suggest that youth with dual-harm constitute a high-risk population and merit particular consideration by researchers and practitioners alike. Because dual-harm is less sex-differentiated than single-harm, prevention and intervention efforts must address both males and females. Protecting children from victimization experiences in the home and peer contexts, improving their sense of social belonging, and supporting their personal resources may alleviate the burden of long-term mental health impairments and anti-social behaviors that youth with dual-harm often experience, and that also negatively affect their families and communities.

## Supplementary material

For supplementary material accompanying this paper visit https://doi.org/10.1017/S0033291722000666. click here to view supplementary material
