# Supplementary material for: Self-, other-, and dual-harm during adolescence: a prospective-longitudinal study of childhood risk factors and early adult correlates
Source: Psychol Med. 2022 Mar 17;53(9):3995–4003. doi: 10.1017/S0033291722000666 (PMC10317800; doi:10.1017/S0033291722000666)
Supplement: Supplementary file 1 [file S0033291722000666sup001.pdf]

Self-, Other-, and Dual-Harm During Adolescence: A Prospective-Longitudinal Study of Childhood Risk Factors and Early Adult Correlates

Annekatrin Steinhoff, Laura Bechtiger, Denis Ribeaud, Manuel Eisner, & Lilly Shanahan

Supplement

Variable descriptions

Table S1. Variables assessing childhood risk factors and early adult correlates

Measure	Number of items, wording, example items	Scale	Cronbach's α	Scoring
**Childhood risk factors (age 7–11)**				
Sensation-seeking, age 7<br><br>Adapted from Alsaker, Naegele, Valkanover, and Hauser (2008)	9 items, assessed using a cardboard game ("travel game") addressing preference for (choice of) sensational versus less sensational situations<br><br>• You must decide whether you want to travel by fast motorbike or funny steam locomotive.	0 = sensational situation not chosen,<br>1 = sensational situation chosen	0.68	Mean score (possible range: 0–1)
Anxiety/depression, age 11<br><br>From the Social Behavior Questionnaire (Murray, Obsuth, Eisner, & Ribeaud, 2019; Tremblay et al., 1991)	8 items<br><br>During the past month, how often have you felt the following?<br>• I had to cry.<br>• I was scared.	1 = never to<br>5 = very often	0.79	Mean score (possible range: 1–5)
Low self-control, age 11<br><br>From the self-control scale (Grasmick, Tittle, Bursik, & Arneklev, 1993)	10 items<br><br>This is about how you see yourself. Mark how true these statements are *for you.*<br>• I often act on the spur of the moment without stopping to think.<br>• If I don't get something I want immediately, I get angry pretty quickly.<br>• I like to get out and do things more than I like to read or contemplate ideas.	1 = fully untrue to 4 = fully true	0.75	Mean score (possible range: 1–4)
Relational aggression (perpetration), age 11	3 items<br><br>Please indicate which of	1 = never to<br>5 = very often	0.76	Mean score (possible range: 1–5)

From the Social Behavior Questionnaire (Murray, Obsuth, et al., 2019; Tremblay et al., 1991)	these things you have done in the last 6 months.<br>• When you were mad at another kid, you said bad things about him/her behind the kid's back.<br>• When you were mad at another kid, you got others to dislike that kid as well.			
Physical aggression (perpetration), age 11<br><br>From the Social Behavior Questionnaire (Murray, Obsuth, et al., 2019; Tremblay et al., 1991)	3 items<br><br>Please indicate which of these things you have done in the last 6 months.<br>• You physically attacked other people.<br>• You got into fights.	1 = never to 5 = very often	0.76	Mean score (possible range: 1–5)
Any substance use, age 11	3 items<br><br>In the last year, have you…<br>• smoked cigarettes?<br>• smoked a joint (hashish, marijuana, or cannabis)?<br>• drunk at least one glass of alcohol (e.g., beer, vodka, mixed drinks, wine)?	0 = no<br>1 = yes	--	Binary:<br>0 = no substance use,<br>1 = any substance use
Delinquency, age 11<br><br>(Eisner, Manzoni, & Ribeaud, 2000; Ribeaud & Eisner, 2009; Wetzels, Enzmann, Mecklenburg, & Pfeiffer, 2001), adapted and expanded	8 items<br><br>In the last year, have you…<br>• Stolen something at home?<br>• Stolen something from a shop or kiosk that is worth more than 50 CHF?<br>• Used a bus, tram, or train without having a valid ticket?	0 = no<br>1 = yes	--	Sum score (possible range: 0–8)
Parental divorce, age 11	1 item<br><br>Parent divorced by child age 11.	0 = no<br>1 = yes	--	Binary:<br>0 = no,<br>1 = yes
Lack of parental involvement, age 11<br><br>From the Alabama Parenting Questionnaire (Shelton, Frick, & Wootton, 1996)	6 items<br><br>Please mark how often the things below occur.<br>• Your parents talk to you about your friends or about the other students in your class.<br>• Your mother or father hugs you to comfort you when you are sad.	1 = never to 4 = often	0.66	Binary coding indicating belonging to top 25% on the mean scale distribution (possible range: 1–4):<br>0 = lower 75%,<br>1 = top 25%

Harsh parenting, age 11<br><br>From the Alabama Parenting Questionnaire (Shelton et al., 1996)	5 items<br><br>When you misbehave or are disobedient, do your parents…<br>• spank you with their hand?<br>• pull your hair or ears?<br>• yell or scream at you?	1 = never to<br>4 = often/always	0.66	Binary coding indicating belonging to top 25% on the mean scale distribution (possible range: 1–4):<br>0 = lower 75%,<br>1 = top 25%
Assault victimization, age 11<br><br>Adapted from (Wetzels et al., 2001)	2 items<br><br>In the past 12 months, have any of the things below happened to you?<br>• Someone purposely injured you with a weapon (e.g., a knife) or object (e.g., a cane) or by repeatedly kicking you with heavy shoes.<br>• Someone hit you so hard that they injured you (e.g. drawing blood or causing a black eye) without using a weapon or objects.	0 = no, 1 = yes	--	Binary:<br>0 = none,<br>1 = any
Bullying victimization, age 11<br><br>From the Zurich Brief Bullying Scale (Murray, Eisner, et al., 2019)	4 items<br><br>In the past 12 months, how often have other people purposely…<br>• ignored or excluded you?<br>• laughed at, mocked, or insulted you?	1 = never to<br>6 = (almost) every day	0.72	Mean score (possible range: 1–6)
Low school bonding, age 11<br><br>Created by study team	9 items<br><br>You've been to school for over four years now. How are you doing there? Please tell us how true the statements below are for you.<br>• I enjoy going to school.<br>• My teacher treats me fairly.<br>• We have a really good sense of community within the class.	1 = fully untrue to 4 = fully true	0.80	Mean score (possible range: 1–4)

Adult correlates (age 20)				
Anxiety/depression<br><br>From the Social Behavior Questionnaire (Murray, Obsuth, et al., 2019; Tremblay et al., 1991)	13 items<br><br>During the past month, how often have you felt the following?<br>(age 20)<br>• I didn't feel joy about anything.<br>• I cried.<br>• I was scared for no particular reason.	1 = never to 5 = very often	0.92	Mean score (possible range: 1–5)
Low self-control<br><br>From the self-control scale (Grasmick et al., 1993)	10 items<br><br>This is about how you see yourself. Mark how true these statements are *for you.*<br>• I often act on the spur of the moment without stopping to think.<br>• If I don't get something I want immediately, I get angry pretty quickly.<br>• I like to get out and do things more than I like to read or contemplate ideas.	1 = fully untrue to 4 = fully true	0.74	Mean score (possible range: 1–4)
Relational aggression (perpetration)<br><br>From the Social Behavior Questionnaire (Murray, Obsuth, et al., 2019; Tremblay et al., 1991)	4 items<br><br>Please indicate which of these things you have done in the last 6 months.<br>• When you were mad at someone, you said bad things about him/her behind the kid's back.<br>• When you were mad at someone, you got others to dislike that kid as well.	1 = never to 5 = very often	0.73	Mean score (possible range: 1–5)
Physical aggression (perpetration)<br><br>From the Social Behavior Questionnaire (Murray, Obsuth, et al., 2019; Tremblay et al., 1991)	3 items<br><br>Please indicate which of these things you have done in the last 6 months.<br>• You physically attacked other people.<br>• You got into fights.	1 = never to 5 = very often	0.85	Mean score (possible range: 1–5)
Psychopathy<br><br>From The Short Dark Triad (Jones & Paulhus, 2013)	6 items<br><br>This is about how you see yourself. Mark how true these statements are *for you.*<br>• People always regret messing with me.<br>• It is true that I can be mean to others.	1 = fully untrue to 4 = fully true	0.73	Mean score (possible range: 1–4)

Suicidal ideation<br><br>From the Violent Ideations Scale (Murray, Eisner, & Ribeaud, 2018)	1 item<br><br>How often have you thought about killing yourself in the last month?	1 = never to<br>5 = very often	--	Binary:<br>0 = never,<br>1 = at least once
Homicidal ideation<br><br>From the Violent Ideations Scale (Murray et al., 2018)	1 item<br><br>How often have you thought about killing someone else you know in the past month?	1 = never to<br>5 = very often	--	Binary:<br>0 = never,<br>1 = at least once
Frequent or illicit substance use<br><br>(Quednow et al., 2021; Shanahan et al., 2021)	14 items<br><br>How often did you take the following substances during the past 12 months?<br><br>• Beer, wine, alcopops<br>• Liquor<br>• Cannabis<br>• Ecstasy<br>• Cocaine<br>• Amphetamines	1 = never,<br>6 = 53–365 times (daily)	--	Binary:<br>0 = no illicit drug use, no daily alcohol use, and no weekly or daily cannabis use, 1 = any illicit drug use or daily alcohol use or weekly or daily cannabis use
Delinquency<br><br>(Eisner et al., 2000; Ribeaud & Eisner, 2009; Wetzels et al., 2001), adapted and expanded	23 items<br><br>In the last year, have you…<br>• Stolen something at home?<br>• Stolen something from a shop or kiosk that is worth more than 50 CHF?<br>• Used a bus, tram, or train without having a valid ticket?<br>• Driven after consuming alcohol or other drugs?<br>• Urged somebody to engage in sexual acts?	0 = no, 1 = yes	--	Sum score (possible range: 0–23)
Assault victimization<br><br>Adapted from (Wetzels et al., 2001)	2 items<br><br>In the past 12 months, have any of the things below happened to you?<br>• Someone purposely injured you with a weapon (e.g. a knife) or object (e.g. a cane) or by repeatedly kicking you with heavy shoes.<br>• Someone hit you so hard that they injured you (e.g.	0 = no, 1 = yes	--	Binary:<br>0 = none,<br>1 = any

	drawing blood or causing a black eye) without using a weapon or object.			
Bullying victimization<br><br>From the Zurich Brief Bullying Scale (Murray, Eisner, et al., 2019)	4 items<br><br>In the past 12 months, how often have other people purposely…<br>• ignored or excluded you?<br>• laughed at, mocked, or insulted you?	1 = never to 6 = (almost) every day	0.63	Mean score (possible range: 1–6)
Perceived social exclusion<br><br>from (Bude & Lantermann, 2006)	6 items<br><br>This is about how you see yourself opposite other people and society. What do you think about the following statements?<br>• I feel like I'm not really part of society.<br>• I don't have a chance in this society anyway.<br>• I feel surplus to society.	1 = fully untrue to 4 = fully true	0.88	Mean score (possible range: 1–4)

Socio-demographic characteristics of dual-, single-, and no harm groups

Variable descriptions

Socio-demographic variables were sex (1 = male, 0 = female), parental education background (1 = at least one parent with a tertiary educational degree, 0 = both parents with a lower or no degree), parental migration background (1 = both parents born abroad, 0 = at least one parent born in Switzerland), and child's educational level at age 13 (1 = high, 0 = low). In Switzerland, children are tracked into different educational levels at ages 12/13 and 15/16. Here we use an indicator of whether the respondents followed an academic track by age 13 (called "high") or a lower track.

Associations with dual- and single-harm between ages 13 and 17

Sex differences in dual- and single-harm are described in the main manuscript. Parental educational background was not associated significantly with either dual-harm or other-harm only. However, low parental educational background was associated with a higher risk of self-harm only (17.6% of those with a low parental educational background reported self-harm only, compared to 12.6% of those with a high parental educational background; OR = 1.49, 95% CI = 1.01–2.18, p = 0.042). Parental migration background was not associated with dual- or single-harm. Dual-harm was more prevalent among adolescents following a lower educational track at age 13 compared to those in the higher track (8.8% vs. 6.0%; OR = 1.51, 95% CI = 1.01–2.25, p = 0.043). Single-harm was not significantly related to child educational level.

Table S2. Adjusted associations between childhood risk factors (independent variables) and single-harm between ages 13 and 17 (dependent variable): Results from multinomial regression analyses (OR [95% CI]) controlling for sex, parental educational and migration background, and child's educational level at age 13.

**Childhood risk factors: independent variables**	**Self-harm only vs. other-harm only**	**Self-harm only vs. no harm**	**Other-harm only vs. no harm**
Sensation-seeking	0.93 (0.73–1.18)	**1.21** (1.02–1.44)	**1.31** (1.08–1.59)
Anxiety/depression	1.10 (0.88–1.39)	**1.28** (1.09–1.50)	1.16 (0.95–1.41)
Lack of self-control	**0.80** (0.65–0.99)	1.17 (1.00–1.38)	**1.46** (1.22–1.76)
Relational aggression	1.12 (0.89–1.40)	**1.24** (1.06–1.46)	1.12 (0.93–1.34)
Physical aggression	**0.76** (0.59–0.98)	**1.27** (1.04–1.55)	**1.67** (1.38–2.01)
Substance use	0.49 (0.20–1.19)	1.00 (0.47–2.11)	**2.04** (1.17–3.54)
Delinquency	**0.80** (0.65–0.98)	1.12 (0.95–1.33)	**1.40** (1.18–1.65)
Parental divorce	0.98 (0.59–1.61)	**1.52** (1.05–2.21)	**1.56** (1.04–2.33)
Lack of parental involvement	1.30 (0.76–2.21)	**1.89** (1.28–2.78)	1.45 (0.94–2.25)
Harsh parenting	0.85 (0.52–1.40)	1.37 (0.91–2.07)	**1.62** (1.07–2.44)
Assault victimization	1.01 (0.61–1.66)	**1.78** (1.23–2.57)	**1.77** (1.17–2.66)
Bullying victimization	1.17 (0.96–1.44)	**1.41** (1.20–1.65)	**1.20** (1.01–1.43)
Lack of school bonding	0.87 (0.69–1.08)	**1.20** (1.01–1.43)	**1.39** (1.16–1.67)

Note. A separate regression model was specified for each correlate. Bold print indicates significant effects ($p < 0.05$).

Table S3. Adjusted associations between single-harm (ages 13–17; independent variables) and mental health and social impairments in early adulthood (age 20; dependent variables): Results from linear regression analyses (a: β, p) and binary logistic regression analyses (b: OR, 95% CI) controlling for sex, parental educational and migration background, and child's educational level at age 13.

**Early adulthood correlates: dependent variables**	**Self-harm only vs. other-harm only**	**Self-harm only vs. no harm**	**Other-harm only vs. no harm**
Anxiety/depression (a)	**0.10** (0.016)	**0.16** (< 0.001)	0.05 (0.104)
Lack of self-control (a)	0.02 (0.585)	**0.11** (< 0.001)	**0.08** (0.005)
Relational aggression (a)	−0.01 (0.861)	0.06 (0.080)	0.06 (0.053)
Physical aggression (a)	**−0.12** (0.005)	0.03 (0.268)	**0.13** (< 0.001)
Psychopathy (a)	−0.07 (0.077)	**0.10** (0.001)	**0.17** (< 0.001)
Suicidal ideation (b)	**2.47** (1.37–4.44)	**2.78** (1.88–4.11)	1.13 (0.67–1.88)
Homicidal ideation (b)	0.86 (0.39–1.93)	1.31 (0.69–2.47)	1.52 (0.85–2.72)
Frequent/illicit substance use (b)	1.32 (0.85–2.05)	**2.15** (1.54–3.01)	**1.63** (1.15–2.31)
Delinquency (a)	−0.02 (0.714)	**0.11** (< 0.001)	**0.12** (0.001)
Assault victim (b)	0.85 (0.40–1.81)	1.73 (0.89–3.35)	**2.04** (1.10–3.79)
Bullying victim (a)	−0.01 (0.806)	**0.08** (0.011)	**0.08** (0.015)
Perceived social exclusion (a)	**0.11** (0.011)	**0.16** (< 0.001)	0.05 (0.107)

Note. A separate regression model was specified for each correlate. Bold print indicates significant effects ($p < 0.05$).

Dual-and single-harm and change of psychological and social functioning from childhood to early adulthood

Table S4. Adjusted associations between dual-/single-/no harm (ages 13–17; independent variables) and mental health and social impairments between childhood and early adulthood (age 20; dependent variables): Results from linear regression analyses (a: *β*, *p*) and binary logistic regression analyses (b: OR, 95% CI) controlling for sex, parental educational and migration background, child's educational level at age 13, and corresponding childhood indicator of outcome (to indicate change in respective outcome).

**Early adulthood correlates: dependent variables**	**Dual-harm vs. no harm**	**Dual-harm vs. self-harm only**	**Dual-harm vs. other-harm only**	**Self-harm only vs. other-harm only**	**Self-harm only vs. no harm**	**Other-harm only vs. no harm**
Anxiety/depression (a)	**0.17** (< 0.001)	0.07 (0.062)	**0.14** (< 0.001)	**0.10** (0.021)	**0.14** (< 0.001)	0.04 (0.150)
Lack of self-control (a)	**0.10** (0.001)	0.03 (0.350)	0.06 (0.102)	0.04 (0.301)	**0.10** (0.001)	0.05 (0.072)
Relational aggression (a)	**0.10** (0.003)	0.07 (0.060)	0.06 (0.128)	−0.01 (0.747)	0.04 (0.171)	0.05 (0.071)
Physical aggression (a)	**0.16** (< 0.001)	**0.14** (0.001)	0.07 (0.127)	**−0.10** (0.016)	0.02 (0.415)	**0.11** (0.001)
Frequent/illicit substance use (b)	**2.52** (1.57–4.04)	1.16 (0.71–1.91)	1.63 (0.94–2.83)	1.40 (0.90–2.19)	**2.17** (1.55–3.02)	**1.55** (1.09–2.20)
Delinquency (a)	**0.14** (< 0.001)	0.07 (0.069)	0.07 (0.083)	0.00 (1.00)	**0.10** (< 0.001)	**0.10** (0.005)
Assault victim (b)	**4.64** (2.48– 8.67)	**2.79** (1.33–5.82)	**2.36** (1.16–4.82)	0.85 (0.40–1.81)	1.67 (0.85–3.27)	**1.96** (1.04–3.68)
Bullying victim (a)	**0.13** (0.001)	**0.09** (0.038)	0.07 (0.090)	−0.02 (0.649)	**0.06** (0.064)	**0.07** (0.032)

Note. A separate regression model was specified for each correlate. Bold print indicates significant effects ($p < 0.05$). To avoid multi-collinearity, we did not enter all risk factors simultaneously into the models.

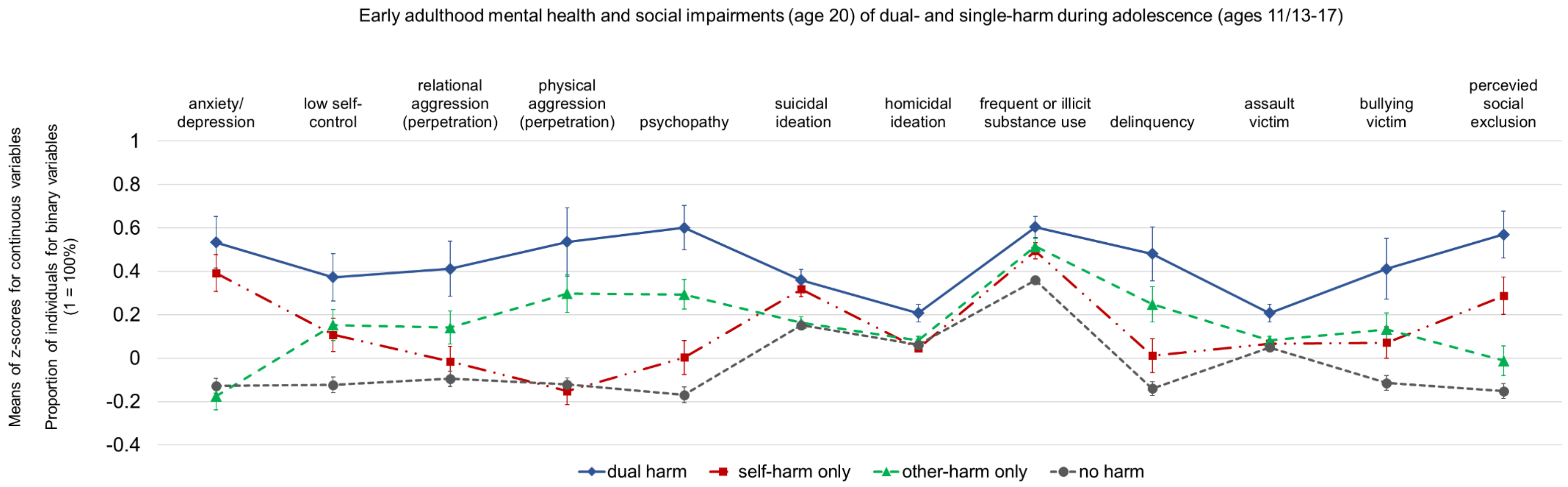

Figure S1. Sensitivity analysis of early adulthood correlates of dual- and single-harm, with age-11 other-harm being included in the coding of harm groups.

References

Alsaker, F. D., Naegele, C., Valkanover, S., & Hauser, D. (2008). *Pathways to victimization and a multisetting intervention: Project documentation*.

Bude, H., & Lantermann, E. D. (2006). Soziale Exklusion und Exklusionsempfinden [Social exclusion and feeling socially excluded]. *Kölner Zeitschrift für Soziologie und Sozialpsychologie, 25*, 233-252.

Eisner, M., Manzoni, P., & Ribeaud, D. (2000). *Gewalterfahrungen von Jugendlichen. Opfererfahrungen und selbstberichtete Gewalt bei Schülerinnen und Schülern im Kanton Zurich*. Aarau: Sauerlaender.

Grasmick, H. G., Tittle, C. R., Bursik, R. J., & Arneklev, B. J. (1993). Testing the core empirical implications of Gottfredson and Hirschi's General Theory of Crime. *Journal of Research in Crime and Delinquency, 30*(1), 5-29. doi:10.1177/0022427893030001002

Jones, D. N., & Paulhus, D. L. (2013). Introducing the Short Dark Triad (SD3) - a brief measure of dark personality traits. *Assessment, 21*(1), 28-41. doi:10.1177/1073191113514105

Murray, A. L., Eisner, M., & Ribeaud, D. (2018). Development and validation of a brief measure of violent thoughts: the Violent Ideations Scale (VIS). *Assessment, 25*(7), 942-955. doi:10.1177/1073191116667213

Murray, A. L., Eisner, M., Ribeaud, D., Kaiser, D., McKenzie, K., & Murray, G. (2019). Validation of a brief self-report measure of adolescent bullying perpetration and victimization. *Assessment*, 1073191119858406. doi:10.1177/1073191119858406

Murray, A. L., Obsuth, I., Eisner, M., & Ribeaud, D. (2019). Evaluating longitudinal invariance in dimensions of mental health across adolescence: An analysis of the Social Behavior Questionnaire. *Assessment, 26*(7), 1234-1245. doi:10.1177/1073191117721741

Quednow, B. B., Steinhoff, A., Bechtiger, L., Ribeaud, D., Eisner, M., & Shanahan, L. (2021). High prevalence and early onsets: legal and illegal substance use in an urban cohort of young adults in Switzerland. *European Addiction Research*. doi:10.1159/000520178

Ribeaud, D., & Eisner, M. (2009). *Entwicklung von Gewalterfahrungen Jungendlicher im Kanton Zurich*. Oberentfelden: Sauerlaender.

Shanahan, L., Steinhoff, A., Bechtiger, L., Copeland, W. E., Ribeaud, D., Eisner, M., & Quednow, B. B. (2021). Frequent teenage cannabis use: Prevalence across adolescence and associations with young adult psychopathology and functional well-being in an urban cohort. *Drug and Alcohol Dependence, 228*. doi:10.1016/j.drugalcdep.2021.109063

Shelton, K. K., Frick, P. J., & Wootton, J. (1996). Assessment of parenting practices in families of elementary school-age children. *Journal of Clinical Child Psychology, 25*(3), 317-329. doi:10.1207/s15374424jccp2503_8

Tremblay, R. E., Loeber, R., Gagnon, C., Charlebois, P., Larivee, S., & LeBlanc, M. (1991). Disruptive boys with stable and unstable high fighting behaviour patterns during junior elementary school. *Journal of Abnormal Child Psychology, 19*, 285-300.

Wetzels, P., Enzmann, D., Mecklenburg, E., & Pfeiffer, C. (2001). *Jugend und Gewalt: eine repraesentative Dunkelfeldanalyse in Muenchen und acht anderen deutschen Staedten* (Nomos Ed.). Baden-Baden.
